# Rare pediatric tumors in Germany – not as rare as expected: a study based on data from the Bavarian Cancer Registry and the German Childhood Cancer Registry

**DOI:** 10.1007/s00431-022-04484-x

**Published:** 2022-04-27

**Authors:** Aisana Achajew, Ines B. Brecht, Martin Radespiel-Tröger, Martin Meyer, Markus Metzler, Claudia Bremensdorfer, Claudia Spix, Friederike Erdmann, Dominik T. Schneider, Michael Abele

**Affiliations:** 1 Department of Internal Medicine, Hospital Fürth, Fürth, Germany; 2 Pediatric Hematology/Oncology, Department of Pediatrics, University Hospital Tuebingen, Tuebingen, Germany; 3 Bavarian Cancer Registry, Bavarian State Office for Health and Food Safety Nuremberg, Nuremberg, Germany; 4 Department of Pediatrics, University Hospital Erlangen, Erlangen, Germany; 5 German Childhood Cancer Registry, Division of Childhood Cancer Epidemiology, Institute for Medical Biostatistics, Epidemiology, and Informatics, University Medical Center Mainz, Mainz, Germany; 6 Department of Prevention and Evaluation, Leibniz Institute for Prevention Research and Epidemiology, Bremen, Germany; 7 Clinic of Pediatrics, Municipal Hospital Dortmund, University Witten/Herecke, Dortmund, Germany

**Keywords:** Rare tumors, Pediatric cancers, Cancer registry, Bavaria, Germany, Incidence

## Abstract

Very rare pediatric tumors (VRTs) pose a challenge for treating physicians as little is known about the best diagnostic assessment and therapeutic decision-making in these malignancies. A large proportion of these cancers occur in adolescence. Therefore, the established structures of pediatric oncology including cancer registration may partly be circumvented. This may lead to an underregistration in clinical cancer registries of yet unclear extent. The aim of this study is to increase the knowledge on the occurrence of VRTs in pediatric patients in Germany. Pseudonymized data of cases recorded in the Bavarian Cancer Registry (BCR) between 2002 and 2014 were retrieved. VRTs according to the definition of the European Cooperative Study Group for Pediatric Rare Tumors were identified using the ICD and ICD-O classification. The numbers of registered patients were compared to those reported to the German Childhood Cancer Registry (GCCR). 6.3% (*n* = 290) of all malignancies (*n* = 4615) in the age below 18 years were classified as VRTs. Median age at diagnosis was 15 years (range 0–17 years). The most common tumor types included malignant melanoma, skin carcinoma, and gonadal tumors. During the same period, 49 pediatric patients from Bavaria with matchable VRTs were reported to the GCCR, accounting for 17% of cases reported to the BCR.

*Conclusions*: The frequency of VRTs in Germany is underestimated in the national GCCR. With this study, we present population-based data on the incidence of VRTs in Germany for the first time. In order to gain additional knowledge about these malignancies, registration of VRTs must be improved through enhanced data exchange between the GCCR, the public cancer registries, and the clinical Registry for Rare Pediatric Tumors (STEP).

**Table:** 

**What is Known:** *• Rare pediatric tumors pose a challenge for treating physicians as limited knowledge is available on these malignancies for diagnostic and therapeutic decision-making.* *• Little is known about the frequency of these rare tumors in pediatric patients.*	
**What is New:** *• The frequency of rare pediatric tumors in Germany is distinctly underestimated in the German Childhood Cancer Registry.* *• We present population-based data on the incidence of these rare pediatric cancers for the first time.*

**What is Known:**

*• Rare pediatric tumors pose a challenge for treating physicians as limited knowledge is available on these malignancies for diagnostic and therapeutic decision-making.*

*• Little is known about the frequency of these rare tumors in pediatric patients.*

**What is New:**

*• The frequency of rare pediatric tumors in Germany is distinctly underestimated in the German Childhood Cancer Registry.*

*• We present population-based data on the incidence of these rare pediatric cancers for the first time.*

## Introduction

About 2200 children and adolescents < 18 years with diagnoses of malignant diseases and central nervous system (CNS) tumors are reported to the German Childhood Cancer Registry (GCCR) every year [1]. According to the RARECARE definition classifying tumors with an incidence rate of < 60/1,000,000 per year as rare, all childhood malignancies would have to be considered rare diseases [2, 3]. Despite this, the treatment of childhood cancer is highly standardized. The German Society for Pediatric Oncology and Hematology (GPOH) and other international study groups ensure the implementation of clinical trials and the development of specific treatment guidelines. Thereby, over 90% of all children with malignancies in Germany are treated according to standard therapy protocols and are enrolled in clinical treatment trials whenever possible. This has led to a remarkable improvement in prognosis with a 15-year overall survival (OS) of 82% nowadays [1].

While many of those entities occur more frequently, several cancer types belong to the heterogeneous group of very rare tumors (VRT) with an incidence rate of < 2/1,000,000 per year or a lack of entity-specific pediatric studies as defined by the European Cooperative Study Group for Pediatric Rare Tumors (EXPeRT) [3]. Estimations of the occurrence of these VRTs are available from several countries. However, the exact incidence rate remains difficult to determine [4, 5]. Previous analyses mainly determined the proportions of VRTs in relation to all childhood malignancies instead of stating incidence rates. As inclusion criteria of different registers regarding age and tumor entities vary, the comparability of proportions is limited to some extent. An analysis of the GCCR over a 10-year period showed that only 1.2% of all registered patients met the EXPeRT definition of a VRT [6]. But the authors already concluded that numbers were probably underestimated due to a lack of registration of specific diagnoses. The Italian Pediatric Rare Tumor Group (TREP) estimated a proportion of VRTs in childhood between 8 and 10% of all pediatric cancers [5]. An analysis of the American Surveillance, Epidemiology, and End Results (SEER) registry estimated that 8% of cancer patients under the age of 15 years and 14% of cancer patients under the age of 20 years were diagnosed with an entity classifying as a VRT according to the EXPeRT definition [4]. With regard to these varying reports on the occurrence of VRTs in childhood, the aim of the present analysis was to assess the degree of underregistration and estimate a more realistic incidence rate of rare pediatric tumors in Germany.

## Materials and methods

We obtained data on pediatric cancer cases from the Bavarian Cancer Registry (BCR), a population-based cancer registry of the second-largest federal state of Germany with approximately 13 million inhabitants. Cancer registration in Germany is conducted by population-based public cancer registries on the level of federal states according to the Federal Cancer Registry Data Act. The registration completeness is estimated to be ≥ 90% since 2003 [7]. In Bavaria, hospital physicians, registered doctors, dentists, and pathologists are entitled to pass the patients’ data on to their respective regional clinical cancer registries, which transfer the recorded data to a central confidentiality office where the data is pseudonymized and finally passed on to the overarching registration office in the BCR [8]. Herein, all malignant neoplasms are recorded as well as all CNS tumors and tumors of borderline histology. It is assumed that the occurrence of childhood cancer in Bavaria is representative for Germany as the respective incidence rates of childhood cancer do not differ significantly between Bavaria and the rest of Germany [1].

All patients registered within the BCR meeting the following inclusion criteria were included in the analysis: diagnosis of malignant disease with the codes of the International Classification of Diseases (ICD) C00–C97, first diagnosis at age < 18 years, and diagnosis between 2002 and 2014. Pseudonymized data was additionally derived from the registry for patient-related data (sex, month and year of birth, municipality code) and tumor-related data (month and year of diagnosis, age at diagnosis, cancer site (ICD) and histology per International Classification of Diseases for Oncology (ICD-O), status per TNM Classification of Malignant Tumors (TNM), grading).

For the extraction of patients with VRTs, we applied the definition of the EXPeRT group: “any solid malignancy or borderline tumor characterized by an annual incidence < 2/million and/or not already considered in clinical trials” [9]. The respective entities were selected using ICD, ICD-O, and the International Classification of Childhood Cancer (ICCC) based on the consensus listing of rare pediatric cancers as well as the entity-specific pediatric studies of the GPOH [3, 10]. Duplicate reports could be excluded by rechecking specifications like municipality code, diagnosis, month and year of diagnosis, and month and year of birth.

The nationwide German Childhood Cancer Registry (GCCR) records incident cases of all malignancies as well as non-malignant CNS tumors diagnosed in 0- to 17-year olds in Germany, reported by all pediatric hematology-oncology units in Germany (subject to the patient’s or custodians consent). Before 2009, only patients aged < 15 years were recorded by the GCCR. The analysis and reporting of childhood cancer incidence rate estimates in Germany are usually based on data from the GCCR [1, 11].

Patients with the same combination of age, sex, year of diagnosis, and diagnostic group as well as residence in Bavaria in both databases were identified and numbers were compared. Partially, the distribution of ICD-O morphology codes for similar cases differed slightly. For our evaluation, we used the ICD-O codes of the BCR as this registry was our primary data source.

For a small number of patients (*n* = 45/4615), the respective cancer entity was not defined clearly in the BCR as either ICD or ICD-O was missing; these cases were excluded from our analysis.

We calculated crude incidence rates, determined as the annual number of cases per person-years calculated as the average population count between 2002 to 2014. Bavarian childhood population estimates were obtained from the Bavarian State Office for Statistics.

## Results

Between 2002 and 2014, the BCR recorded 4615 children diagnosed with malignancy at ages 0–17 years with a median age at diagnosis of 9 years. This corresponds to an average annual crude incidence rate of 160 per 1,000,000 children of this age group. Crude incidence rates by diagnostic group are presented in Table 1.

**Table 1:** Frequencies and crude incidence rates of childhood cancer diagnosis groups according to the ICCC [10], registered by the Bavarian Cancer Registry 2002–2014

	0 to 17 years			< 10 years	10 to 17 years
	Count	Proportion of cases	Annual incidence rate per million	Count	Count
I. Leukemias, myeloproliferative, and myelodysplastic diseases	1236	26.8%	42.8	900	336
II. Lymphomas and reticuloendothelial neoplasms	699	15.1%	24.2	224	475
III. CNS and miscellaneous intracranial and intraspinal neoplasms^a^	840	18.2%	29.1	485	355
IV. Neuroblastoma and other peripheral and intraspinal neoplasms	236	5.1%	8.2	217	19
V. Retinoblastoma	54	1.2%	1.9	54	0
VI. Renal tumors	191	4.1%	6.6	175	16
VII. Hepatic tumors	57	1.2%	2.0	49	8
VIII. Malignant bone tumors	281	6.1%	9.7	63	218
IX. Soft tissue and other extraosseous sarcomas	266	5.8%	9.2	130	136
X. Germ cell tumors, trophoblastic tumors, and neoplasms of gonads^a^	223	4.8%	7.7	64	159
XI. Other malignant epithelial neoplasms and malignant melanomas	488	10.6%	16.9	43	445
XII. Other and unspecified malignant neoplasms	44	1.0%	1.5	21	23
**Total**	**4615**	**100%**	**160.0**	**2425**	**2190**

^a^Excluding non-malignant diagnoses

**Fig. 1:**
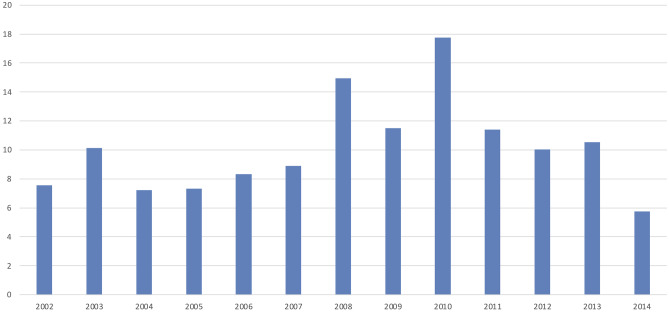
Annual cases of very rare pediatric tumors per million inhabitants aged 0–18 years registered with the Bavarian Cancer Registry 2002–2014

We identified 990 patients (21.5% of all malignancies) with a cancer type that is estimated to have an incidence rate lower than 2 per 1 million or classifies as an orphan disease without consideration in entity-specific studies. Out of these, 290 cases (6.3% of all malignancies) could not be enrolled within an entity-specific study or registry of the GPOH as no such study or registry was available and thus are considered rare in the sense of the EXPeRT definition. This corresponds to a crude annual incidence rate of all pediatric VRTs in Bavaria of 10.1 per million. The diagnostic groups and specific cancer types of these cases are shown in Table 2. The most common tumor types among VRTs were malignant melanoma (*n* = 134, 46.2%) followed by the group of other malignant epithelial neoplasms (*n* = 94, 32.4%). The frequency of patients with VRTs was relatively stable over the observed time period of 13 years as displayed in Fig. 1.

**Table 2:** Frequencies and crude incidence rates of rare childhood cancers according to the EXPeRT definition [3], registered by the Bavarian Cancer Registry 2002–2014

	0 to 17 years			< 10 years	10 to 17 years
	Count	Proportion of cases	Annual incidence rate per million	Count	Count
**IV. Neuroblastoma and other peripheral and intraspinal neoplasms**					
Esthesioneuroblastoma	2	0.7%	0.1	1	1
**VIII. Malignant bone tumors**					
Chondrosarcoma	9	3.1%	0.3	2	7
Chondroblastoma (malignant)	1	0.3%	0.0	0	1
Adamantinoma of long bones	3	1.0%	0.1	1	2
Malignant chordoma	5	1.7%	0.2	3	2
Malignant fibrous neoplasms of bone	2	0.7%	0.1	0	2
**X. Germ cell tumors, trophoblastic tumors, and neoplasms of gonads**					
Gonadal carcinomas	18	6.2%	0.6	0	18
Granulosa cell tumor	8	2.8%	0.3	2	6
Sertoli Leydig tumor	7	2.4%	0.2	1	6
Sex cord stromal tumor, not further specified	2	0.7%	0.1	1	1
**XI. Other malignant epithelial neoplasms and malignant melanomas**					
Nasopharyngeal carcinomas	6	2.1%	0.2	0	6
Carcinoma of bladder	4	1.4%	0.1	0	4
Carcinoma of cervix uteri	14	4.8%	0.5	1	13
Carcinoma of salivary glands	9	3.1%	0.3	1	8
Carcinomas of colon and rectum	9	3.1%	0.3	0	9
Carcinomas of lung	1	0.3%	0.0	0	1
Carcinomas of other specified sites	17	5.9%	0.6	1	16
Carcinomas of unspecified site	2	0.7%	0.1	1	1
Malignant melanoma	134	46.2%	4.6	13	121
Pancreatic carcinoma	1	0.3%	0.0	0	1
Skin carcinoma	28	9.7%	1.0	5	23
Solid pseudopapillary neoplasms	3	1.0%	0.1	0	3
**XII. Other and unspecified malignant neoplasms**					
Other specified malignant tumors	4	1.4%	0.1	1	3
Epithelioid malignant mesothelioma	1	0.3%	0.0	0	1
**Total**	**290**	**100%**	**10.1**	**34**	**256**

A tumor was considered rare if it fulfills the following criteria: annual incidence < 2/1,000,000 or status as an orphan disease, which is not considered in any clinical study or registry of the GPOH. The different diagnosis groups were formed according to the ICCC [10]

Among VRTs registered to the BCR, 34 (11.7%) patients were younger than 10 years and 256 (88.3%) older than 10 years. Accordingly, the median age at the time of diagnosis of a pediatric VRT was 15 years. The age distribution was similar among other rare pediatric tumors with an annual incidence rate of < 2 per million which were included in entity-specific studies or registries (< 10 years: 32.9%; ≥ 10 years: 67.1%). In contrast, the more frequent entities, characterized by an annual incidence rate of > 2 per million were predominantly diagnosed in children < 10 years (59.9%).

Malignant melanoma was the most common tumor type in both age groups followed by skin carcinomas. In children ≥ 10 years, the third frequent subgroup was gonadal carcinomas. The male to female ratio was 1:1.17. Malignant melanoma was the most common entity in both genders, followed by various carcinoma and gonadal tumors. At diagnosis, advanced disease stages of VRTs were rarely detected. In most reported cases, there were no lymph node metastases (85.2%) and distant metastases (94.9%) at diagnosis. However, data on TNM staging was missing in about 40% of cases in the recordings of the BCR.

In the same time period and with similar inclusion criteria, the number of registered cases of VRTs in the GCCR was considerably lower than that in the BCR. The GCCR reported 49 cases of VRTs in Bavaria, which corresponds to 16.9% of the patients with VRTs recorded in the BCR. While the GCCR did not record patients > 15 years before 2009, the proportion recorded in the registered cases remains the same for the time span 2009–2014 (15.5%), when patients with cancer diagnoses at age 15–17 years were included in the registry. The GCCR reported 22 cases of VRTs in Bavaria between 2009 and 2014, whereas the BCR recorded 142 cases of VRTs during the same period. Further details on this comparison are presented in Table 3. When comparing proportions of cases reported to the GCCR and BCR, 44.1% of VRT cases in the BCR in the age group < 10 years were recorded with the GCCR, while only 13.2% of VRT cases in the BCR in the age group ≥ 10 years were recorded with the GCCR. The discrepancy between the respective recording frequencies may at least in part be explained by the coverage of more cases belonging to ICCC-3 class XI “Other malignant epithelial neoplasms and malignant melanomas” in the BCR (*n* = 125) than in the GCCR (*n* = 14). Furthermore, we found slightly higher counts for category VIII (“Malignant bone tumors”) and category X (“Germ cell tumors, trophoblastic tumors, and neoplasms of gonads) in Bavaria. When comparing numbers by diagnosis, we found that malignant melanoma and other malignant skin cancers were the most common missing cases in the GCCR.

**Table 3:** Frequencies of rare childhood cancers aged 0–17 years according to the EXPeRT definition [3], registered by the Bavarian Cancer Registry (BCR) and the German Childhood Cancer Registry (GCCR) 2009–2014

	BCR—Count	GCCR—Count
**IV. Neuroblastoma and other peripheral and intraspinal neoplasms**		
Esthesioneuroblastoma	0	0
**VIII. Malignant bone tumors**		
Chondrosarcoma	6	1
Chondroblastoma (malignant)	0	0
Adamantinoma of long bones	1	0
Malignant chordoma	3	3
Malignant fibrous neoplasms of bone	1	0
**X. Germ cell tumors, trophoblastic tumors, and neoplasms of gonads**		
Gonadal carcinomas	10	1
Granulosa cell tumor	2	2
Sertoli Leydig tumor	1	0
Sex cord stromal tumor, not further specified	1	0
**XI. Other malignant epithelial neoplasms and malignant melanomas**		
Nasopharyngeal carcinomas	2	1
Carcinoma of bladder	2	0
Carcinoma of cervix uteri	10	0
Carcinoma of salivary glands	4	0
Carcinomas of colon and rectum	3	1
Carcinomas of lung	1	0
Carcinomas of other specified sites	18	4
Carcinomas of unspecified site	1	1
Malignant melanoma	61	5
Pancreatic carcinoma	0	0
Skin carcinoma	20	0
Solid pseudopapillary neoplasms	3	2
**XII. Other and unspecified malignant neoplasms**		
Other specified malignant tumors	41	0
Epithelioid malignant mesothelioma	1	1
**Total**	**142**	**22**

## Discussion

We identified 290 cases of rare childhood cancers according to the EXPeRT definition in Bavaria with diagnosis between 2002 and 2014, corresponding to 6.3% of all registered malignancies in the BCR and a crude annual incidence rate of all VRTs of 10.1 per million. This compares to an analysis of the American SEER registry, which found a crude annual incidence rate of all VRTs of 21.3 per million and estimated that 14% of cancer cases under 20 years belonged to rare entities. This difference could likely be attributed to the inclusion of older age groups in the SEER cohort as the most common pediatric VRTs occur with older age [4]. Furthermore, the SEER analysis used a different definition of VRTs than our analysis, as it includes tumors that are classified as very rare only in the age group 0–14 years but with a higher frequency in the age group 0–19 years. In addition, some VRTs are recorded in entity-specific studies in Germany despite their rarity but are not included in the SEER analysis. Thus, tumors like hepatoblastoma, hepatic carcinoma, thyroid carcinoma, and Ewing sarcoma were included in the SEER analysis. Therefore, these entities contributed substantially to the higher incidence rate of VRTs in the SEER analysis compared to our analysis. However, the incidence of VRTs registered at the BCR might still rise to a certain extent during the next years, even if the registration completeness has already met ≥ 90%. Nevertheless, reporting cancer diagnoses to the BCR has become mandatory by law only in 2017 and as a result registration may still increase [7]. However, a limitation of our analysis has to be considered, as VRTs in the BCR had specific ICD-O morphology codes but possible changes in diagnosis at a later time after initial registration may not always have been recorded in the BCR.

The numbers of detected cases of VRTs in the BCR were set against cases registered in the GCCR in the same time period. When comparing the registration rates for VRTs not reported to clinical studies or registries, there was a significant registration gap between the BCR and the GCCR, which was most evident among adolescent VRT patients. In fact, only 49 of the 290 cases registered in the BCR were also reported to the GCCR. This significant registration gap is most likely due to differences in the registration structure between the GCCR and the BCR. In the BCR, all cancer cases in Bavaria were reported by treating physicians as well as pathologists, irrespective of age and treating department. In contrast, the GCCR only received notifications from specialized pediatric oncology units and not from hospitals that specialized in cancer care for adult patients. These notifications were limited to patients under the age of 15 years until 2008. Afterwards, the GCCR also recorded reports of cancer patients up to the age of 18 years [1]. However, the inclusion of patients aged 15–17 years in the GCCR did not increase the reporting of VRTs considerably. Accordingly, these patients with VRT do not seem to have been treated in pediatric hematology-oncology units that are used to report to the GCCR.

In accordance with previous studies, our analysis showed that VRTs are more common in adolescents, as 88% occurred in patients aged 10–17 years [3, 6]. As many of these are epithelial neoplasms or gonadal tumors that occur more frequently in adulthood, a significant proportion of the adolescent patients with VRTs may be treated in adult oncological therapy units (e.g. dermatooncology, gynecooncology, ENT oncology, medical oncology) and may thus not be reported to the GCCR. In our comparison of BCR and GCCR data, malignant melanoma and skin carcinoma account for nearly 60% of VRTs and are substantially underreported in the GCCR. Diagnosis in early stages of disease without metastases may have favored the omission of interdisciplinary care of patients including pediatric oncology. When comparing the registration numbers of pediatric malignant melanoma in different registries, the phenomenon of under-registration becomes even more evident. An earlier analysis of the GCCR revealed 55 cases of malignant melanoma with an age < 18 years in Germany over 10 years [6]. Another publication analyzed the German Central Malignant Melanoma Registry (CMMR), which receives data from cooperating dermatology departments and dermatologic practitioners throughout Germany. The CMMR registered 443 pediatric patients ≤ 18 years of age over a time period of nearly 30 years [12]. The comparison of these numbers indicates that an entity such as malignant melanoma, which is common in adults but rare in children, is often treated in adult centers. However, as we found 134 pediatric cases of malignant melanoma registered in 13 years in Bavaria alone, a relevant underregistration has to be postulated not only for the GCCR but also for the CMMR, both being dependent on voluntary registration. Thus, the incidence of childhood malignant melanoma in Germany is likely to be considerably higher than previously described. Similarly, the incidence of other rare pediatric tumors, particularly in adolescence, may be underestimated, as current reports on the occurrence of cancer in childhood in Germany are based on data from the GCCR [11].

Obviously, older children with rare “adult-type” tumors may benefit from the experience of an adult specialist [13]. For example, pediatric patients with malignant melanoma, who were treated by adult dermatologists, had a comparable outcome to adult patients [12]. Besides, pediatric patients with adult cancer types may benefit from early clinical trials in adults, being selected for compassionate use of similar strategies and medications. Nevertheless, regular recording of childhood malignant melanoma in a distinct registry could facilitate the identification of characteristics specific to this age group that could influence age-specific treatment guidelines. Furthermore, several entities, such as colorectal carcinoma, are known to show differences in tumor biology and behavior depending on whether they occur in children or adults [14]. Besides, the occurrence of epithelial malignancies in childhood is more frequently associated with tumor predisposition syndromes. Moreover, long-term treatment-associated morbidity has a much stronger impact on children compared to elderly patients [14]. Therefore, standards of diagnosis and treatment of certain malignancies should not simply be transferred from adults to children [15]. It is important to develop close collaboration between pediatric and adult specialists on certain cancer entities to ensure the best treatment possible for children suffering from rare cancers. The underregistration of patients in the German childhood cancer registry illustrated the need to improve registration structures for children and adolescents in Germany and to strengthen data exchange between adult and pediatric clinical cancer registries as well as epidemiological registries. Based on comprehensive registration, clinical and research networks can be intensified, which will allow patients access to the best possible treatment and clinical research, despite the extreme rarity of their disease.

## Conclusions

We conclude that forces need to be united to enable a better registration of rare pediatric tumor cases. This can be achieved by improved cooperation between distinct pediatric and adult oncological departments within the established comprehensive cancer centers. Thus, registration of all cases of rare cancers in childhood and adolescence shall be ensured independent from the treating department. While German legislation has addressed the issue of regular data exchange between clinical and epidemiological cancer registries in 2021, the new law only requires the development of a joint concept for cooperation between state cancer registries and the GCCR [16]. Furthermore, a nationwide mandatory reporting of all childhood cancers should be established to optimize recording and close registration gaps. This will be the prerequisite for a better understanding of rare entities, further research, the establishment of clinical trials, and the development of evidence-based pediatric guidelines for diagnosis and treatment of these tumors. Realizing the importance of improved care for rare cancers in children, the German Registry for Rare Pediatric Tumors (STEP) and the EXPeRT group developed an interdisciplinary network of childhood and adult cancer experts on a national and international level. This network will ensure the optimal treatment of rare pediatric tumors according to the latest clinical experiences and research findings.

## Data Availability

The data that support the findings of this study are available on request from the corresponding author, [MA]. The data are not publicly available due to privacy restrictions.

## Abbreviations

BCR: Bavarian Cancer Registry
CNS: Central nervous system
EXPeRT: European Cooperative Study Group for Pediatric Rare Tumors
GMMR: German Central Malignant Melanoma Registry
GCCR: German Childhood Cancer Registry
GPOH: German Society for Pediatric Oncology and Hematology
ICCC: International Classification of Childhood Cancer
ICD: International Classification of Diseases
ICD-O: International Classification of Diseases for Oncology
OS: Overall survival
SEER: Surveillance, Epidemiology, and End Results registry
STEP: German Registry for Rare Pediatric Tumors
TNM: TNM Classification of Malignant Tumors
TREP: Italian Pediatric Rare Tumor Group
VRT: Very rare pediatric tumor
